# Joint Spreading Factor and Channel Assignment in Multi-Operator LoRaWAN Deployments

**DOI:** 10.3390/s21010162

**Published:** 2020-12-29

**Authors:** Hassan Fawaz, Kinda Khawam, Samer Lahoud, Cedric Adjih, Steven Martin

**Affiliations:** 1 Institut Mines-Télécom, Université Paris-Saclay, UVSQ, 78035 Versailles, France; Kinda.Khawam@uvsq.fr; 2 Faculty of Engineering ESIB, Université Saint-Joseph de Beyrouth, ESIB, CIMTI, Beirut, Lebanon; samer.lahoud@usj.edu.lb; 3 Inria, CRI Saclay-Île-de-France, 91120 Palaiseau, France; cedric.adjih@inria.fr; 4 Université Paris-Saclay, LRI, 91190 Gif-sur-Yvette, France; steven.martin@lri.fr

**Keywords:** LoRaWAN, game theory, spreading factor, channel selection

## Abstract

LoRaWAN is a popular internet of things (IoT) solution over the unlicensed radio band. It sustains low-cost, durable, and long range IoT wireless communications. Nonetheless, with over 24 billion connected IoT devices being expected by the end of the year, and over 50 billion by 2025, the concurrent and legacy approaches to spreading factor and channel assignment in LoRaWAN networks can no longer keep up. This is exacerbated with the growing densification of IoT device deployments and, with the increasing requirements for better throughput and packet delivery ratios. In this paper, we propose a proportional fair-based joint optimal formulation for spreading factor and channel assignment in multi-operator LoRaWAN deployments. The objective of this problem is to maximize the total sum of the logarithmic normalized throughput. We split the problem into two subproblems, and propose a game theoretic approach to solving them. We prove that our games converge towards a pure Nash equilibrium and, afterwards, solve the optimization problems using both semi-distributed and completely distributed algorithms. Via simulations, we show that our algorithms greatly improve the total normalized throughput for LoRaWAN as well as the packet success rate, in comparison to the legacy approaches.

## 1. Introduction

With an ever increasing demand for reliability within IoT deployments, and with the inevitable densification of internet of things (IoT) devices, concurrent approaches to spreading factor (SF) and channel assignment in Long Rage Wide Area Networks LoRaWAN networks no longer suffice. The low power, long range, and durable transmissions in LoRaWAN take place over the frequency channels of the unlicensed industrial, scientific, and medical (ISM) bands while using a spread-spectrum modulation. Six spreading factors that range from SF7 to SF12 are used. Transmissions that are on the same SFs and on the same frequency channels lead to collisions. The latter become inescapable with multiple LoRaWAN deployments cohabiting the same areas. A 1% duty cycle limitation, which was introduced to maintain fairness in the ISM band, does little to improve the reliability of LoRaWAN networks, while simultaneously limiting their scalability.

In this paper, we aim to tackle the various problems facing the reliability and scalability of LoRaWAN via a joint SF and channel assignment proposal. We start by presenting an optimal formulation for the joint problem aimed at optimizing the throughput in a fair manner. Because of the complexity of the problem, and its unattainable requirement of a centralized solver in a multi-operator deployment, we choose to divide the problem into two: (a) an SF assignment subproblem and (b) a channel assignment subproblem. We use a non-cooperative game theory approach in order to portray each subproblem. The different operators are *competing, yet rational*, players of these games, and they each seek to maximize their own utility.

For the SF assignment subproblem, we propose a semi-distributed best response algorithm aimed at finding the Nash equilibrium (NE) of the game. We additionally propose a heuristic to make it completely distributed.

For the channel assignment subproblem, we show that our game possesses a weighted potential function and that, by extension, a pure NE exists and it can be reached via both best response and replicator dynamics. The game permits selecting the number of channels to be allocated to each operator, with its traffic being equally distributed on the channels it selects.

We solve the decoupled problem for SF assignment first, and for channel assignment second. The joint algorithm can be semi-distributed (best response approaches) or completely distributed (learning approach). We describe our proposals and then compare them to the concurrent approach in SF and channel selection in LoRaWAN, which consists of adaptive data rate (ADR) for SF selection, along with random channel selection for each packet.

The rest of this paper is structured, as follows: Section 2 describes the related works from the state-of-the-art and our contributions. In Section 3, we introduce our system model. We discuss our optimal formulation for SF and channel assignment in Section 4. Our game for SF assignment is detailed in Section 5, and its counterpart to channel assignment in Section 6. Section 7 presents the simulations and results. Finally, the paper is concluded with Section 8.

## 2. Related Works and Contributions

In this section, we highlight some of the state-of-the-art approaches to SF and channel assignment in LoRaWAN and discuss how our proposals differ from them.

We start with some influential publications on the capabilities and limitations of LoRaWAN. Using an ns3 simulator, the authors of [1] analyze the scalability of legacy LoRaWAN. They illustrate how downlink traffic negatively impacts the packet delivery ratio and conclude that duty cycle limitations hinder the scalability of LoRaWAN. In [2], the authors study the legacy ADR SF selection algorithm and deduce that, while it does reduce device energy consumption, it does also limit the expansion of LoRaWAN networks. When considering ISM band interferences, the authors of [3] study their impact on the bit error rate. They prove that there exists a signal-to-interference ratio threshold beyond which the impact of narrow-band interference becomes negligible.

More related to the context of our work in this paper, the authors in [4], propose what they labeled as a *smart* spreading factor selection approach for LoRaWAN networks. They aimed to optimize the SF selection process while using support vector machines and decision tree classifier machine learning. Their simulation results show that their algorithm improves the packet delivery ratio for LoRaWAN.

In [5], an interference-aware algorithm for SF selection is proposed. Several factors are taken into consideration by the authors, including the sensitivity of the gateways and the interfering SFs. They propose an algorithm that decreases the time-on-air for every device in the network. They illustrate how their algorithm increases the total packet delivery ratio in comparison to the legacy LoRaWAN SF selection approach.

The authors of [6] propose a traffic oriented SF selection algorithm that equalizes the traffic loads on the SF channels. They additionally propose a K-means algorithms with the objective of relieving the crowded regions that are suffering from excessive collisions. The authors show that their proposed algorithms help to improve the scalability of LoRaWAN networks.

With the objective of increasing the data delivery ratio in LoRaWAN, the authors of [2,7] detail the limitations of the legacy SF selection algorithm and, thereafter, propose algorithms to tackle them. In [2], the authors argue that their approach can improve the scalability of LoRaWAN with a trade-off of a marginal increase in device power consumption. In [7], the authors claim that their proposed algorithm can improve data delivery in a dense LoRaWAN deployment.

An extension to the legacy ADR approach, which is called ExpLoRa-SF, is presented in [8]. ExpLoRa-SF equally distributes the SFs on the devices subject only to device SNR. Through simulations, they show that their proposal can outperform the legacy approach.

In relation to the ISM band channels, the authors in [9] present a channel control scheme for LoRa. They balance the traffic on all available channels. They show how their proposal outperforms legacy and state-of-the-art proposals in dense deployment scenarios.

In [10], the authors present a scheduling algorithm that aims to ameliorate the scalability of LoRaWAN. Their proposal schedules the SFs, the channels, and the time slots for all the devices-to-gateway wireless links. They show the gains in packet delivery ratios from their proposal in comparison to the Aloha access scheme.

The authors in [11] study the performance of a Multi-Armed Bandit algorithm for channel selection in LoRaWAN. An analysis of the proposal simulation results show that each device ends up selecting the appropriate channel based on what improves its packet delivery ratio.

Finally, in [12] the authors present a lightweight scheduling algorithm for SF and channels the selection in LoRaWAN networks. Their proposal, RS-LoRa, lets devices choose their own SFs according to probability distributions defined in response to the observed rate following the utilization of each SF. In a single cell scenario with 1000 devices present, they show that their proposal can reduce the packet error rate by 20% with respect to legacy LoRaWAN networks.

In this paper, we consider multiple co-located network operators, unlike the vast majority of the state-of-the-art that work on single-cell, single operator networks [2,4,5,7,9,10,12]. This invalidates many of the state-of-the-art proposals that presume that their algorithms can be addressed in a centralized manner, an unrealistic assumption in a multi-operator deployment. Our proposals compute solutions at the different operator levels with the results relayed back to the devices during their listening windows. This means that our proposals can be implemented for all LoRa device classes, unlike many state-of-the-art algorithms that require device-level intelligence [11,12]. Additionally, our work does not rely on classical scheduling, as in [10], an unrealistic approach with a 1% duty cycle limitation in place.

We summarize our contributions, as follows:

(a) We propose an optimal formulation for SF and channel assignment in a multi-operator LoRaWAN deployment. We divide the problem into two: (a) an SF assignment subproblem and (b) a channel assignment subproblem. We propose non-cooperative games for each subproblem, which we solve successively until reaching their pure Nash equilibrium.
(b) We propose a semi-distributed approach for finding the NEs of the decoupled problem based on best response dynamics.
(c) After proving their convergence, we propose a replicator dynamics based learning approach for finding the NE for the channel selection game. We thereafter propose a distributed approach for solving the decoupled problem.
(d) We simulate our proposals and compare them to the concurrent approach to SF and channel selection in LoRaWAN networks. We highlight the gains that they bring in terms of throughput, packet delivery ratios, and energy efficiency.

## 3. System Model and Specifications

**Figure 1:**
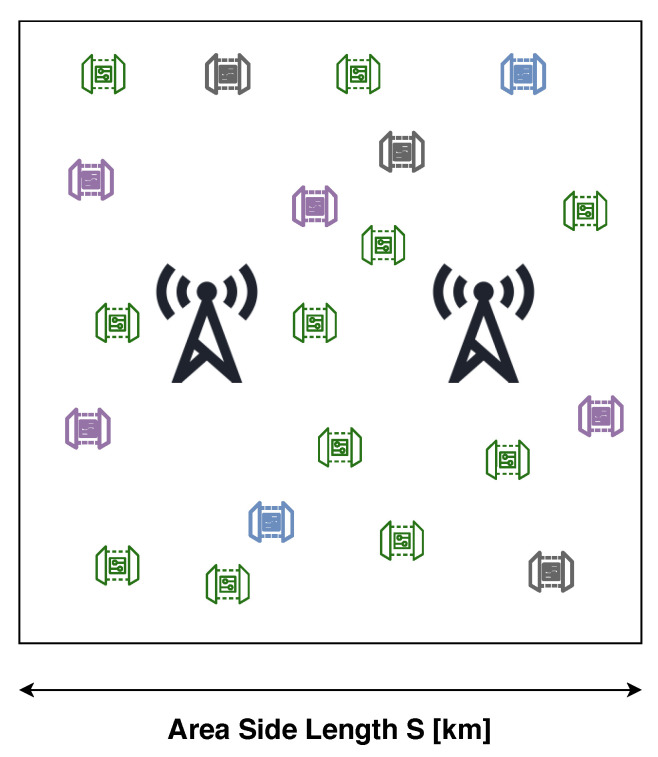
A scenario with two LoRaWAN gateways and four operators.

We consider multiple LoRaWAN deployments belonging to different operators. Each network *i*∈N has $N^{i}$ nodes and $r^{i}$ gateways. The gateways for the different operators are coincident, i.e., they are present at the same location sites. The devices are scattered across a square shaped area, as shown in Figure 1.

All of the nodes generate packets with a rate $\lambda^{i}$. Transmission attempts are done according to a Poisson process with packet size *l* being constant. Let $T_{s}$ be the time that is needed to transmit a packet of size *l* while using spreading factor *s*. LoRa supports SFs that range from 7 to 12. The latter represent a trade-off between coverage and data rate. For instance, SF7 yields the highest data rate, but it covers the shortest distance. The SFs are orthogonal meaning that packets transmitted during the same time frame with different SFs will be successfully received. LoRa utilizes forward error correction to detect and correct transmission errors with the coding rate set to 4/(*C*+4), where *C*∈ {1,2,3,4}. Table 1 shows the variation of data rate, sensitivity, and SNR thresholds as a function of the SFs that are utilized in the 868 MHz band for *C* = 4.

**Figure 2:**
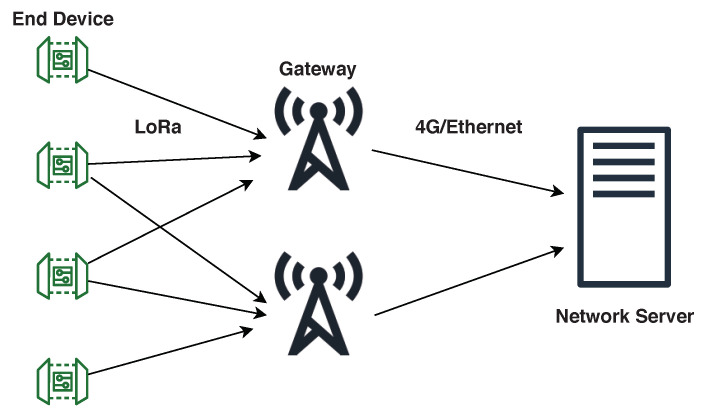
LoRaWAN architecture.

The upper layer protocol LoRaWAN uses a star topology. Each network connects to its own network server with one hop communications, where all of the gateways forward the packets to the server. Figure 2 presents a typical architecture of such a network.

Because LoRaWAN uses pure Aloha as a channel access scheme, the duty cycle in the LoRa band is limited to *d* = 1%. As such, the packet generation rate must verify that λ$T_{s}$ ≤ *d*. LoRaWAN supports multiple frequency bands within the unlicensed bands around 433, 868, and 915 MHz. In Europe, the 868 MHz band is used with 125, 250, and 500 kHz bandwidth channels.

**Figure 3:**
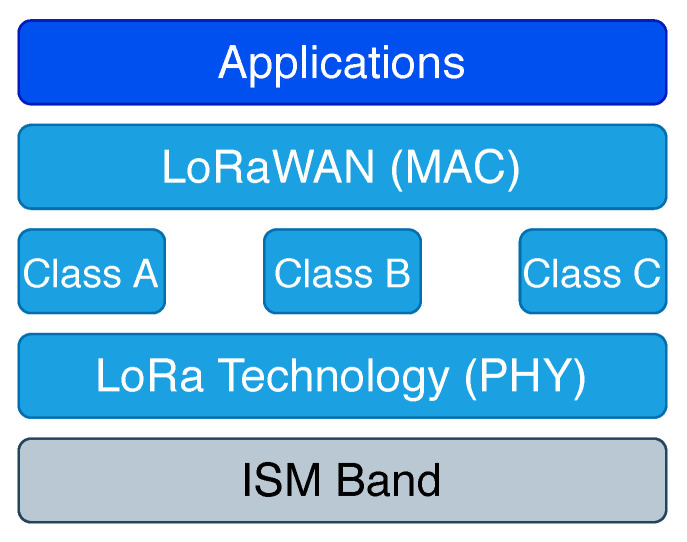
LoRaWAN node network stack.

Figure 3 shows a typical network stack of a LoRa node. LoRa devices can be divided into three classes based on the application:

- Class A: the device wakes up to send a packet. After sending the packet, the device listens for a short period of time before going back to sleep. This class of LoRa devices consumes the least amount of energy.
- Class B: the device has regularly scheduled listening periods.
- Class C: under this configuration, a device is always listening. This leads to maximum energy consumption.

An additional advantage for our proposals in this paper is that, contrary to many others in the state-of-the-art, they do not require additional synchronization to be added to current LoRaWAN specifications for class A. The network server can notify the nodes of their SF and channel assignments within their receiving windows. Finally, Table 2 has a summary of the notations concerning our system model.

## 4. Optimal Formulation for SF and Channel Assignment

We consider multiple co-located operators. Suppose that the different operator devices are transmitting on one of a set of available channels C. We introduce the normalized channel traffic load per SF, and per channel $G_{sc}$, as:

(1) $$G_{sc}=\lambda_{sc}^{e}\cdot T_{s}+\sum_{i=1}^{N}\frac{1}{n}\cdot x_{i}^{c}\left(\lambda^{i}\cdot N_{co}^{i}\cdot p_{s}^{i}\cdot T_{s}\right),$$

where $x_{i}^{c}$ is a binary variable that is equal to one if network operator *i* is using channel *c*, and zero otherwise, and *n* is the number of channels that each operator can use. The total normalized traffic load for a network *i*, per SF, and per channel it is utilizing, can be written as:

(2) $$G_{sc}^{i}=\frac{1}{n}\left(\lambda^{i}\cdot N_{co}^{i}\cdot p_{s}^{i}\cdot T_{s}\right).$$

As such, with the success rate being expressed as $\exp(-2G_{sc})$, the total normalized throughput in the network becomes:

(3) $$T=\sum_{i=1}^{N}\sum_{c=1}^{C}\sum_{s=1}^{S}x_{c}^{i}\cdot G_{sc}^{i}\exp\left(-2G_{sc}\right).$$

We define the proportional fair global optimal problem, in order to maximize the total normalized throughput through spreading factor and channel selection, as follows:

(4a) $$\begin{array}{cc} \; & \underset{x_{c}^{i},p_{s}^{i}}{\mathrm{Maximize}}\sum_{i=1}^{N}\sum_{c=1}^{C}\sum_{s=1}^{S}x_{c}^{i}\cdot \log\left(G_{sc}^{i}\exp\left(-2G_{sc}\right)\right)\\ \; & \mathrm{Subject}\;\mathrm{to} \end{array}$$

(4b) $$\begin{array}{cc} \; & \sum_{s=1}^{S}p_{s}^{i}\leq 1,\;\forall i\in N, \end{array}$$

(4c) $$\begin{array}{cc} \; & \sum_{k=1}^{s}p_{k}^{i}\leq \sum_{k=1}^{s}\frac{N_{k}^{i}}{N_{co}^{i}},\;\forall s=1,\ldots ,S,\;\forall i\in N, \end{array}$$

(4d) $$\begin{array}{cc} \; & \sum_{c=1}^{C}x_{c}^{i}\leq n,\;\forall i\in N, \end{array}$$

(4e) $$\begin{array}{cc} \; & x_{c}^{i}\in \left\{0,1\right\},\;\forall i\in N,\;\forall c\in C. \end{array}$$

The function in (4a) is the objective of the problem, in order to maximize the total normalized throughput in the system over all the channels, over all of the spreading factors. The constraints in (4b) ensure that the sum of the ratio on all of the SFs remains less than or equal to one. The constraints in (4c) ensure that no device could be assigned an SF it cannot use. Finally, the constraints in (4d) and (4e) verify that each operator will not use more than *n* channels.

Not only is the problem with this formulation expensive to solve (MILP), it also requires the presence of a centralized server that can solve the problem in one shot. The latter would have to have all of the information regarding all the different operators. This is not a realistic approach. As a consequence, we divide the problem into two sub problems: (a) a spreading factor selection problem and (b) a channel selection problem. We use game theory to solve each of the aforementioned problems at the operator level.

In our work, we decoupled the joint problem and solved for the SFs first, and for the channels second. Iterating the algorithm i.e., solving again for SF assignment after channel assignment and so on does not yield a different result. This is because, for our objective, the SF selection is independent of what channels the devices are using, as attested to in Equation (7). Finally, the joint assignment algorithm can either be semi-distributed, by using the best response algorithms to solve for the NEs, or completely distributed, by using the learning approach.

## 5. Multi-Operator Game for SF Assignment

We first propose a game for SF selection. This game is played among the different network operators under the assumption of previously selected channels per operator. Each network aims to find the SF distribution $p_{s}^{i}$, which maximizes its own normalized traffic throughput. Nonetheless, networks can not do this independently. Otherwise, within the same geographical zone i.e., devices would have the same radio conditions, all of the devices would default to similar SF selections. This would increase the risk of collisions. The situation makes non-cooperative game theory ideal for SF selection in a multi-operator LoRaWAN deployment, where rational players (the operators) are competing for rather contradicting objectives.

### 5.1. Game Formulation

As such, we define a multi-player game $G_{SF}$ between the different present operators. The formulation of this game $G_{SF}=\langle N,\prod_{i}S_{i},U_{i}\rangle$ can be described, as follows:

- A finite set of players i∈N, the set of operators.
- The action of a given player is the percentage of devices allocated any spreading factor *s*, the strategy chosen by an operator *i* is then $p^{i}=\left(p_{1}^{i},\ldots ,p_{6}^{i}\right)$, the percentage of its devices on each spreading factor, where, for example, $p_{1}^{i}$ is the percentage of devices using SF7.
- For each player *i*, the space of pure strategies is $S_{i}$, such that $S_{i}=\left\{p^{i}\in {\left[0,1\right]}^{S}\right|$ (4b) and (4c), ∀s=1,…,S}.
- A set of utility functions $\left(U_{i\in N}\right)$ that quantify players’ profit for a given strategy profile.

### 5.2. Player Utilities

Each player, i.e., network operator, will seek to maximize the logarithmic sum of its own normalized throughput. As attested to in [13], the logarithmic utility function is intimately associated with the concept of proportional fairness. The goal is to strike a good compromise between efficiency (maximizing the log of throughput will increase the throughput) and fairness. The utility per network operator can be expressed as:

(5) $$U_{i}=\sum_{c=1}^{C}\sum_{s=1}^{S}x_{c}^{i}\cdot \log\left(G_{sc}^{i}\exp\left(-2G_{sc}\right)\right),$$

where $\exp(-2G_{sc})$ represents the success ratio per spreading factor (the same for any network *i*) and $G_{sc}^{i}$ represents the traffic load of network *i*. As such each network will seek to maximize its own delivered traffic, regardless of the others.

### 5.3. Best Response

In game theory, a rational solution is one where all of the competing players adhere to a Nash equilibrium (NE) [14]. An NE is a profile of strategies in which no player will take advantage of the others by unilaterally deviating its strategy. Thus, the primary challenge in game theory is to propose algorithms that are capable of reaching such an equilibrium. The simplest of these algorithms are the repeated best response dynamics. Following these dynamics, each player selects the best, and locally optimal, response to other players’ strategies, until the algorithm converges.

In our game G, for every network *i*, $U_{i}$ is strictly concave with respect to $p^{i}$ and continuous in $p^{-i}$ (the strategy of all other players). Hence, a unique NE exists, and it can be reached via the repeated best response dynamics. As such, we implement a best response algorithm, where, in each iteration $t_{b}$, network *i* seeks to find its optimal SF distribution as a response to $p^{-i}\left(t_{b}-1\right)$ by solving the following problem:

(6a) $$\begin{array}{cc} \; & \underset{p_{s}^{i}}{\mathrm{Maximize}}\;\;U_{i}\left(p^{i},p^{-i}\right)\\ \; & \mathrm{Subject}\;\mathrm{to} \end{array}$$

(6b) $$\begin{array}{cc} \; & \sum_{s=1}^{S}p_{s}^{i}\leq 1, \end{array}$$

(6c) $$\begin{array}{cc} \; & \sum_{k=1}^{s}p_{k}^{i}\leq \sum_{k=1}^{s}\frac{N_{k}^{i}}{N_{co}^{i}},\;\forall s=1,\ldots ,S. \end{array}$$

The optimum $p_{s}^{i*}$ must satisfy the Karush–Kuhn–Tucker (KKT) conditions. There exists a unique Lagrange multiplier α, such that:

(7) $$p_{s}^{i*}=\frac{1}{\alpha +2\lambda^{i}N_{co}^{i}T_{s}},$$

(8) $$\alpha (1-\sum_{s=1}^{S}p_{s}^{i})=0.$$

This produces two cases:

- if α = 0, given that $\sum_{s=1}^{S}p_{s}^{i*}\leq 1$, then $p_{s}^{i*}$ = $\frac{1}{2\lambda^{i}N_{c}^{i}T_{s}}$.
- if α ≠ 0, and, given that $\sum_{s=1}^{S}p_{s}^{i*}\leq 1$, then $\sum_{s=1}^{S}\frac{1}{\alpha +2\lambda^{i}N_{c}^{i}T_{s}}$ = 1.

The best response algorithm we implemented is sketched in Algorithm 1.
**Algorithm 1 **Best Response Algorithm for SF Selection1:**Requires:** Maximum tolerance ϵ←$10^{-5}$2:**Input:** Initial SF assignment limits3:**Initialize:**$t_{b}=0$4:**Do**5:     $t_{b}$←$t_{b}$ + 16:     **For** *i* = 1, …, N7:        Solve the problem in (6) for *i*8:        Update $p_{s}^{i}$9:        Compute $\delta^{i}=\left|\right|p_{s}^{i}\left(t_{b}\right)-p_{s}^{i}\left(t_{b}-1\right)\left|\right|$10:     **End For**11:**While** ∃*i* such that $\delta^{i}\geq \epsilon$

### 5.4. Distributed Approach to SF Assignment

In order to make the SF assignment algorithm completely distributed, it is sufficient for any individual operator to have an estimation of the total packet delivery ratio (success rate) at the time they play the game. For simplicity, we assume that the number of devices present in its network is known by each operator. For instance, if 1000 devices are present and transmitting at one packet per hour, the operator expects to receive 1000 packets every hour. If it receives 600, and then it estimates that it has a packet delivery ratio of 0.6. With this estimation, any operator can play the game in a distributed manner, from simple knowledge of its own traffic.

## 6. Multi-Operator Game for Channel Selection

We additionally use non-cooperative game theory for the purpose of channel selection. In this game, the SF assignment is constant, and the channel selection varies.

### 6.1. Game Formulation

We define a multi-player game $G_{c}$ between the different present operators. The formulation of this game $G_{c}=\langle N,\prod_{i}S_{i}^{c},U_{i}^{c}\rangle$ is described, as follows:

- A finite set of players i∈N, the set of operators.
- The action of a given player is the choice of the channel. The strategy chosen by an operator *i* is then $x^{i}=\left(x_{1}^{i},\ldots ,x_{C}^{i}\right)$, with each $x_{c}^{i}$ indicating whether it utilizes channel *c*.
- For each player *i*, the space of pure strategies is $S_{i}^{c}$, such that $S_{i}^{c}=\left\{x^{i}\in {\left\{0,1\right\}}^{C}\right|$ (4d), ∀c=1,…,C}.
- A set of utility functions $\left(U_{i\in N}^{c}\right)$ that quantify players’ profit for a given strategy profile.

### 6.2. Player Utilities

Each player, i.e., network operator, will seek to greedily maximize the sum of its own normalized throughput that was obtained by the Aloha medium access scheme. The utility per network operator can be expressed as:

(9) $$U_{i}^{c}=\sum_{c=1}^{C}\sum_{s=1}^{S}x_{c}^{i}\log\left(G_{sc}^{i}\exp\left(-2G_{sc}\right)\right)$$

where $\exp(-2G_{sc})$ represents the success ratio per spreading factor and per channel in the entire system (and it coincides with the success ratio of any network *i*), and $G_{sc}^{i}$ represents the traffic load of network *i*. Again, each player of the game is seeking, through channel selection, to maximize its own normalized throughput.

### 6.3. Existence of a Nash Equilibrium

We aim to prove that a pure Nash equilibrium does indeed exist for our game proposal. We do this by showing that this game possesses a weighted potential function [15]. For this proof, we assume that *n* = 1, i.e., each operator is only choosing one channel out of the channels available for transmission. We denote $w^{i}=\sum_{s}\left(\lambda^{i}\cdot N_{co}^{i}\cdot p_{s}^{i}\cdot T_{s}\right).$ In order to prove that a Nash Equilibrium exists for this game, we consider j∈N and define the function ϕ, such that:

(10) $$\begin{array}{c} \phi \left(x^{i},x^{-i}\right)=\frac{1}{2}\sum_{c}\left[\left(\sum_{j}w^{j}x_{c}^{j}\right)\times \left(2\lambda_{c}^{e}+\sum_{j}w^{j}x_{c}^{j}\right)\right]\;\\ \;\;\;\;=\frac{1}{2}\left(\sum_{j\neq i}w^{j}x_{c}^{i}+w^{i}x_{c}^{i}\right)\times \left(2\lambda_{c}^{e}+\sum_{j\neq i}w^{j}x_{c}^{j}+w^{i}x_{c}^{i}\right)\;\\ \;+\frac{1}{2}\left(\sum_{j\neq i}w^{j}x_{c^{\prime }}^{i}+w^{i}x_{c^{\prime }}^{i}\right)\times \left(2\lambda_{c^{\prime }}^{e}+\sum_{j\neq i}w^{j}x_{c^{\prime }}^{j}+w^{i}x_{c^{\prime }}^{i}\right) \end{array}$$

Now, suppose that player *i* changes its choice from channel *c* to channel $c^{\prime }$, then

(11) $$\begin{array}{c} 2\phi \left(x^{i},x^{-i}\right)-2\phi \left(x^{{}^{\prime }i},x^{-i}\right)=w^{i}x_{c}^{i}\times \left(2\lambda_{c}^{e}+\sum_{k}w^{k}x_{c}^{k}\right)+w^{i}x_{c}^{i}\times \left(\sum_{k}w^{k}x_{c}^{k}\right)\;\\ -w^{i}x_{c^{\prime }}^{i}\times \left(2\lambda_{c^{\prime }}^{e}+\sum_{k}w^{k}x_{c^{\prime }}^{k}\right)-w^{i}x_{c^{\prime }}^{i}\times \left(\sum_{k}w^{k}x_{c^{\prime }}^{k}\right)=w^{i}x_{c}^{i}\left(2\lambda_{c}^{e}+2\sum_{k}w^{k}x_{c}^{k}\right)-w^{i}x_{c^{\prime }}^{i}\left(2\lambda_{c^{\prime }}^{e}+2\sum_{k}w^{k}x_{c^{\prime }}^{k}\right)\;\\ \;\;=w^{i}U_{i}^{c}\left(x^{i},x^{-i}\right)-w^{i}U_{i}^{c}\left(x^{{}^{\prime }i},x^{-i}\right), \end{array}$$

where $U_{i}^{c}$ is the cost function associated with $U_{i}^{c}$, such that

(12) $$U_{i}^{c}=-\log\left(\pi_{s}w_{s}^{i}\right)+2\sum_{c}x_{c}^{i}\left[\sum_{k}w^{k}x_{c}^{k}+\lambda_{c}^{e}\right]$$

Because a weighted potential function exists for our proposed game, pure Nash equilibriums exist and we prove, via simulations, their efficiency. For *n*> 1, the potential function still stands. Instead of changing the choice from one channel to another, we are changing from one strategy (two or more channels selected) to another.

### 6.4. Best Response Algorithm for Computing the NE

We first propose a semi-distributed approach for computing the NE based on repeated best response dynamics. In every iteration $t_{c}$, a player *i* (network operator) seeks to find its optimal channel selection as a response to $x_{c}^{-i}\left(t_{c}-1\right)$, the decisions of all the other players, by solving the following problem:

(13a) $$\begin{array}{cc} \; & \underset{x_{c}^{i}}{\mathrm{Maximize}}\;\;U_{i}^{c}\left(x^{i},x^{-i}\right)\\ \; & \mathrm{Subject}\;\mathrm{to} \end{array}$$

(13b) $$\begin{array}{cc} \; & \sum_{c=1}^{C}x_{c}^{i}\leq n,\;\forall i\in N, \end{array}$$

(13c) $$\begin{array}{cc} \; & x_{c}^{i}\in \left\{0,1\right\}\;\forall i\in N,\forall c\in C \end{array}$$

The corresponding pseudo-code for our proposal can be seen in Algorithm 2.
**Algorithm 2 **Best Response Algorithm for Channel Selection1:**Requires:** Maximum tolerance ϵ←$10^{-5}$2:**Input:** Initial channel assignment3:**Initialize:**$t_{c}=0$4:**Do**5:     $t_{c}$←$t_{c}$ + 16:     **For** *i*=1, …, N7:        Solve the problem in (13) for *i*8:        Update channel selection9:        Compute $\delta^{i}=\left|\right|x_{c}^{i}\left(t_{g}\right)-x_{c}^{i}\left(t_{g}-1\right)\left|\right|$10:     **End For**11:**While** ∃*i* such that $\delta^{i}\geq \epsilon$

### 6.5. Distributed Learning-Based Approach to Computing the NE

In this section, we propose using replicator dynamics to solve the problem in a completely decentralized manner. Because our game possesses a potential function, the paper in [15] attests that replicator dynamics will converge almost surely to a pure Nash equilibrium. We first solve the problem for *n* = 1, i.e., each operator only chooses one channel for transmissions. The goal is to stochastically learn the best strategy (channel choice). This is done with each operator selecting the channel randomly, with a set of probabilities ($p_{1}^{i},p_{2}^{i},\ldots ,p_{C}^{i}$). This set is maintained by each player *i* and it is iteratively updated until convergence. Let $p_{c}^{i}\left(t\right)$ be the probability that network operator *i* picks channel *c*. The sum of all the probabilities is equal to 1, for each network *i*, i.e, $\sum_{c\in C}p_{c}^{i}\left(t\right)$ = 1 ∀*i*∈N, ∀*t*. At the beginning of the learning process, channels are selected with equal probabilities. At every time step *t*, the network chooses a certain channel that is based on the consecutively updated probabilities and it is subsequently issued a reward. The resulting utility output is a negative real because our objective introduces a logarithmic function, and since our algorithm operates on numerical values of the tenths order. The reward is, as such, evaluated in terms of minimum cost as opposed to maximum profit. Among the different existing time-difference reinforcement learning approaches, we elect to update the probabilities, after any channel selection is made, as follows:

(14) $$p_{c}^{i}\left(t+1\right)=\left\{\begin{array}{c} p_{c}^{i}\left(t\right)+\beta R\left(1-p_{c}^{i}\left(t\right)\right),\;\mathrm{if}\;\theta_{ic}=1\\ p_{c}^{i}\left(t\right)-\beta Rp_{c}^{i}\left(t\right),\;\;\mathrm{otherwise} \end{array}\right.$$

In the function above, $\theta_{ic}$ is a variable that is equal to one if network operator *i* chooses channel *c*, and zero otherwise. β is the learning rate, which is chosen between 0 and 1, and R is the reward. The reward reflects how good the channel selection was and it is computed as:

(15) $$R=1-\frac{U_{i}^{c}}{U_{max}},$$

where $U_{max}$ quantifies an upper bound for the player utility.

The learning rate β controls both the speed with which the algorithm converges towards a preferred channel selection choice for the different networks, as well as the efficiency of the choice. A high value of β would incur quicker decisions, but it might miss the Nash equilibrium and lead to inefficient decisions. Ideally, we want to chose the highest value of β that would always converge towards an efficient Nash equilibrium.

The network needs to know the packet success rate (total packet delivery ratio) in order to estimate the reward. Similar to before, we assume that the network can produce an estimate after a certain period of utilizing the channel. Algorithm 3 depicts the pseudo-code for our proposed learning algorithm for finding the NE.
**Algorithm 3 **Learning the NE for *n* = 1**Input:** Learning rate β∈ [0,1]**Initialize:**$p_{c}^{i}\left(1\right)$←$\frac{1}{C}$, ∀*i*∈N**Do**$t_{l}$←$t_{l}+1$    **For** *i* = 1, …, N       Network *i* selects a channel following $p_{c}^{i}\left(t\right)$       It utilizes the selected channel sufficiently long       to estimate the success rate per SF       Compute R = $1-U_{i}^{c}/U_{max}$       **For** *c*∈C           **If** $\theta_{ic}$== 1              $p_{c}^{i}\left(t_{l}+1\right)\leftarrow p_{c}^{i}\left(t_{l}\right)+\beta R\left(1-p_{c}^{i}\left(t_{l}\right)\right)$           **Else**              $p_{c}^{i}\left(t_{l}+1\right)\leftarrow p_{c}^{i}\left(t_{l}\right)-\beta Rp_{c}^{i}\left(t_{l}\right)$           **End If**       **End For**    **End For****Until** ∃c∈C such that $p_{c}^{i}\left(1\right)$≈ 1, ∀*i*∈N

### 6.6. Generalization of the Learning Problem

The NE can still be attained for n≥1 while using the same replicator dynamics approach. However, instead of updating the probabilities of a certain channel being allocated, the algorithm works on the probabilities of a certain combination of channels being allocated. If the number of channels chosen by each operator *n* is equal to two, and the total number of available channels is eight, then the total number of possible selections is equal to 82 = 28. Every time slot, and for each player, a couple of channels is selected and, afterwards, the probability of selecting the couple is updated, alongside the other probabilities, such that the sum remains equal to one.

## 7. Simulations and Results

In this section, we test the effectiveness of our proposals and compare them to the state-of-the-art in SF and channel assignment for LoRaWAN. We experiment, by simulations, the two approaches of our game theory-based proposals. The first, which we label as the ‘best response’, uses the best response approaches to both SF assignment and channel selection. The second, which we label as ’learning approach’, combines the distributed algorithm for SF assignment and the dynamic replication learning proposal for channel selection. We compare our algorithms to the currently in use state-of-the-art approaches that are represented by ADR for SF selection by the devices, and random channel assignment per packet. Namely, each device transmits on the lowest available SF and on a randomly selected channel at each packet transmission, as in current LoRaWAN deployments.

Table 3 summarizes our simulation parameters. We consider four co-located operators. The number of devices that correspond to each network is ascending and equal to 750, 1000, 1250, and 1500 devices, respectively. The packet generation rate per device also differs from one network to another and increases from one packet per hour to four, for operators one to four, respectively. The parameters remain, as shown in the table, unless they are changed by the simulation scenario.

### 7.1. Impact of the Area Size

**Figure 4:**
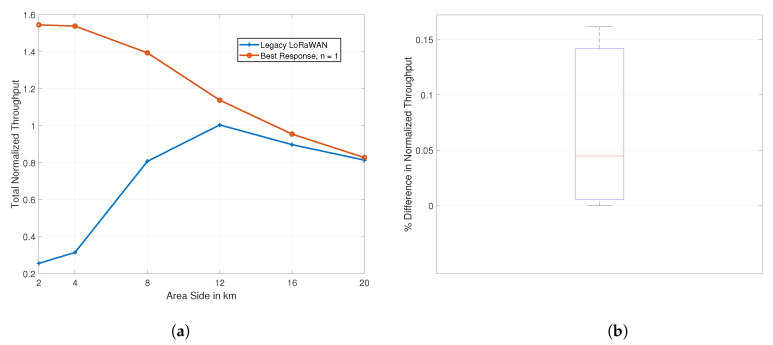
Impact of the area size on the performance. (**a**) Game approach gains. (**b**) Best response vs. learning approach.

We start by studying the impact of the deployment area size on the performance of our game theoretic proposal. We vary the simulation square area size by changing the side length *S* between 2, 4, 8, 12, 16 km, and 20 km. For this simulation, we assume the presence of three channels, with *n* = 1 for our proposal. In other terms, in our algorithm, each operator only chooses one channel. For the legacy approach, all of the channels are open and they are used evenly by the devices. Figure 4 presents the results in terms of the total normalized throughput.

Figure 4a compares the total normalized throughput, across all four networks, between our proposal and the legacy LoRaWAN approach. Our joint SF and channel assignment algorithm acts as an upper bound for the legacy approach. At *S* = 2 km, our proposal results in a total normalized throughput higher than 1.5, as compared to an underwhelming 0.25 for the legacy approach. At *S* = 20 km, both of the algorithms produce throughput values in the vicinity of 0.8 with our proposal ever-so-slightly producing better values.

Both ends of the plot highlight an aspect of legacy LoRaWAN functionality. When the distance between the gateway and the devices is rather small, the majority of the devices using the ADR approach for SF selection will default to SF7. The lower SFs would generally become too crowded, leading to excessive collisions. When the area size is large, the motivations for the legacy approach become visible. The SFs are balanced between the devices and on the different available channels. Nonetheless, our simulation scenario favors the legacy approach in this case. With the densification of IoT devices and gateway deployment, and with the natural need of wireless applications for more reliability, the proportion of devices that are in close proximity to the gateway will increase. Therefore, for most devices, the left side of the graph will be the best indicator of performance.

We additionally assess the performance of our distributed learning approach, in comparison to its semi-distributed best response counterpart. Figure 4b has a box plot of the percentage difference in the total normalized throughput resulting from the use of either of the approaches. The box plot shows insignificant difference in payoffs between the two, with a median difference of less than 0.05% and a maximum difference slightly above 0.15%. This means that the distributed approach could be used to incur less signaling with an insignificant loss in performance.

### 7.2. Impact of Device Densification

**Figure 5:**
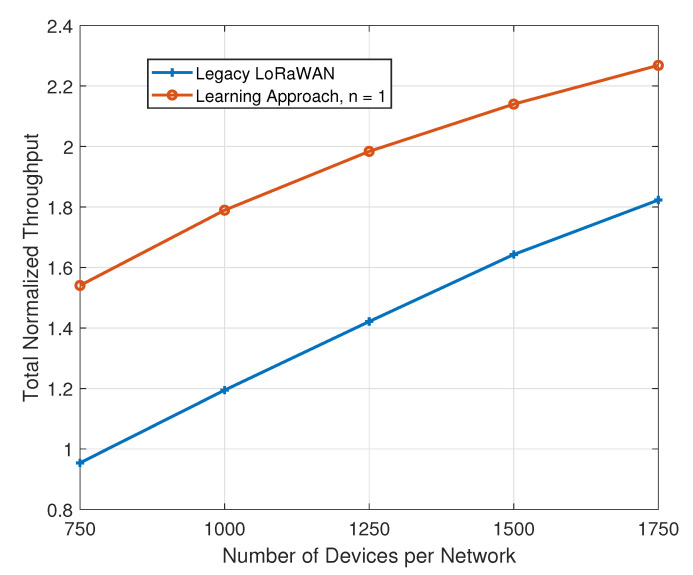
Impact of an increase in the number of devices on the total normalized throughput.

We aim to assess the impact of an increase in the number of IoT devices in the deployment area. To this end, we fix the packet generation rate for all devices, across all networks, at five packets per hour. The number of devices for each network, which we vary in this simulation, is also equal across all networks. The number of available channels is three, and *n* = 1 for our proposal. Figure 5 has a plot with the resulting total normalized throughput values.

Again, we notice a wide gap between our proposal and the legacy approaches to SF and channel assignments. When the number of devices is 750, the total normalized throughput is 0.9542 for the legacy algorithm as compared to 1.54 for our proposal. When the number of devices is 1750, the total normalized throughput is 2.2682 for our proposal, as compared to 1.82 for the legacy approach.

### 7.3. Impact of an Increase in the Number of Available Channels

**Figure 6:**
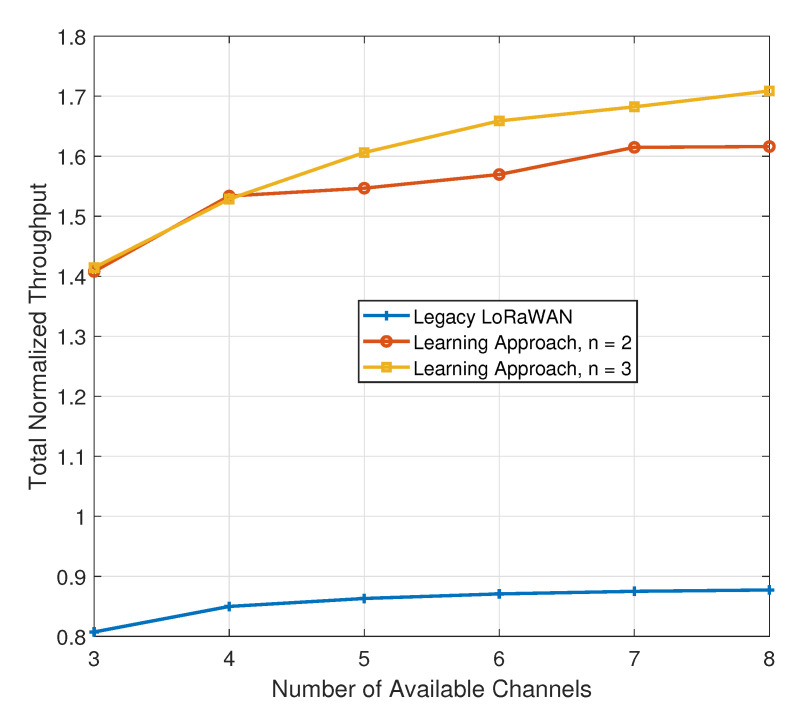
Impact of added channels on the total normalized throughput.

In all subsequent simulation scenarios, we increase the number of available channels from three to eight, the maximum possible under ISM band regulations. With an increase in the number of available channels, and with the legacy algorithm being able to access all of them, we increase the number of channels that each operator chooses in our approach (*n*) from one to two/three. We first study the impact of this increase on the total normalized throughput. The number of available channels is increased gradually from three to eight, which is the LoRaWAN standard. Figure 6 has a plot with the results.

As in previous simulations, our proposal, this time represented by the learning approach, vastly outperforms the legacy algorithm. For a small number of channels (3–4), the value of *n* has no effect on the performance of our algorithm. Beyond that, allowing each operator to choose three channels instead of two further improves the performance. At eight available channels, the legacy LoRaWAN algorithm, which has equal access to all eight, produces a total normalized throughput of less than 0.9. In comparison, our algorithm for *n* = 2 produces a value of 1.616 and for *n* = 3 produces a value close to 1.7.

**Figure 7:**
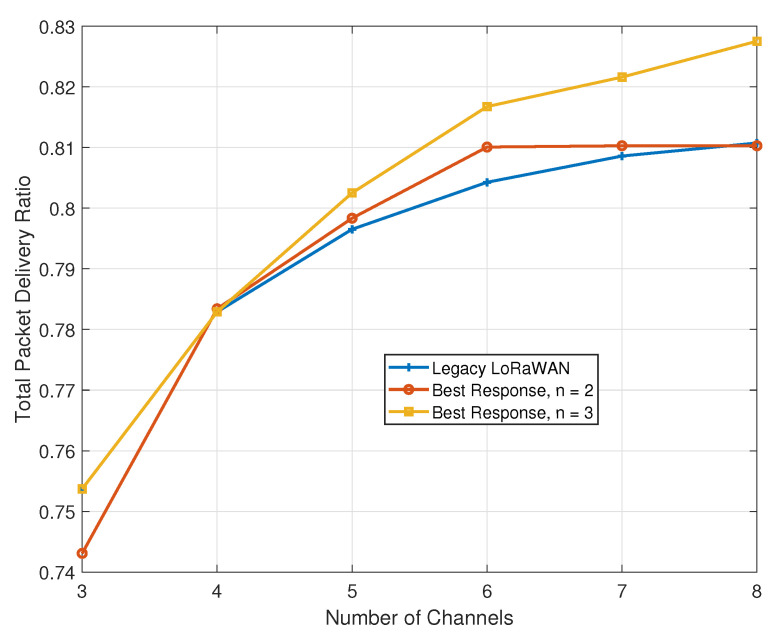
Impact of added channels on the total packet delivery ratio.

We further study the performance of our algorithm in terms of the total packet delivery ratio. Figure 7 presents a plot with the results.

Even though the objective of our problem revolves around maximizing the throughput, our algorithm also has the capability to slightly improve the total packet delivery ratio (success rate). With the exception of the case of three available channels (for *n* = 2), our proposal will regularly outperform the legacy approach. In this scenario, the limited improvement is mainly due to the dense deployment of devices. Across varying scenarios, in terms of the number of devices, the total area size, and the packet generation rates, our proposal can only further widen the performance gap with respect to the legacy approach.

### 7.4. Energy Consumed per Delivered Byte

**Figure 8:**
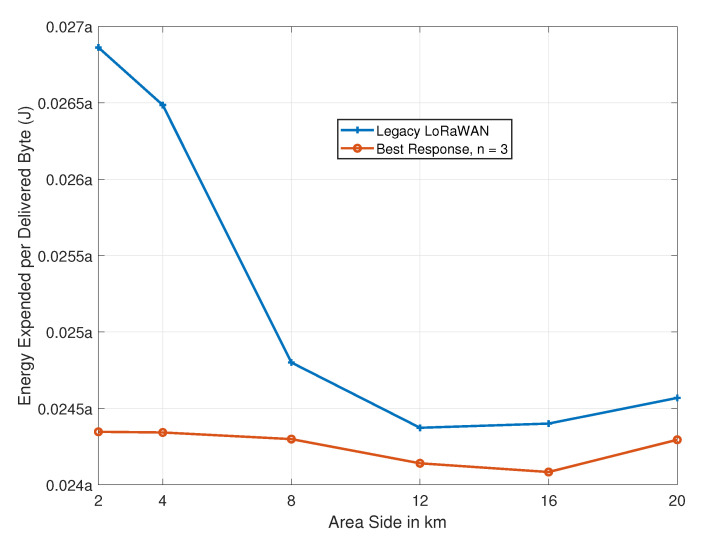
Energy expended per byte as a function of *S*.

We evaluate the average energy that is expended per successful delivery for our proposed algorithm and compare it to the state-of-the-art approach. We consider eight available channels with *n* = 3 for our proposal. Again, the legacy algorithm assumes equal access to all eight channels. We vary the area side length *S* between 2 and 20 km. According to I noticed some errors in the number format in the picture. Is it necessary to modify it [16], the energy cost of data delivery can be expressed as:

(16) $$EC_{delivery}\approx \frac{a\cdot \exp(2G_{sc})}{l_{pay}},$$

where *a* is the energy consumed per transmission attempt, $G_{sc}$ is the normalized traffic load per spreading factor, per channel, and $l_{pay}$ is the payload size. Figure 8 presents the results.

Following the improvement that it brings in terms of packet delivery ratios, our algorithm reduces the energy consumption. For the simulated scenario, up to 10% reduction in energy consumption is possible, owing to our proposal. In other terms, our proposal can simultaneously improve the throughput while reducing power consumption at the devices.

### 7.5. Study of Individual Network Performances

**Figure 9:**
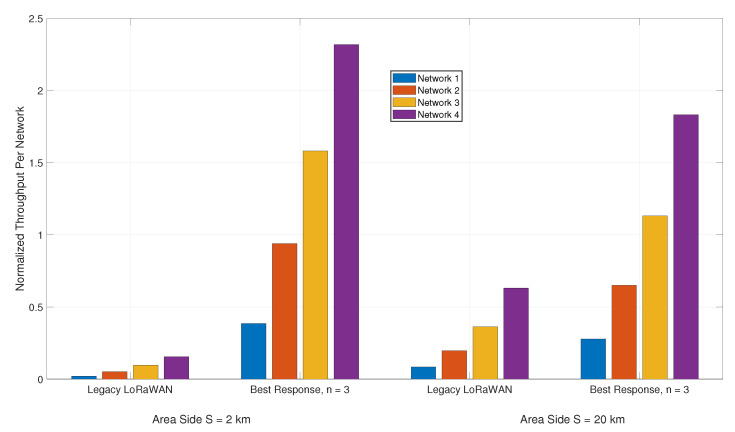
Normalized throughput per network as a function of *S*.

As the players of our game proposals each seek to greedily and selfishly improve their own performance, we inspect the resulting normalized throughput per network. We compare between our proposal (*n* = 3) and the competing LoRaWAN approach. Recall that the networks produce different volumes of traffic with network 1 being the smallest and network 4 the largest. In Figure 9, we compare the resulting network normalized throughput values for *S* = 2 km and *S* = 20 km, the worst and best case scenarios for the legacy approach, as illustrated in Figure 4a, respectively.

The bar plot illustrated in Figure 9 shows that the ability of the legacy approach to close the gap in performance with respect to our proposal at large values of *S*, no longer exists when n=3. At *S* = 2 km, the largest network normalized throughput for the legacy algorithm is 0.1552, noticeably lower than the lowest attained network throughput from our proposal at 0.3859. While this difference in performance does indeed shrink when *S* = 20 km, it remains rather significant. Across all of the networks, our proposal improves the individual network normalized throughput by three times, on average.

## 8. Conclusions

In this paper, we proposed a joint optimal formulation for spreading factor and channel assignment in multi-operator LoRaWAN deployments. The joint problem is decoupled into a set of two games: (a) a spreading factor assignment game and (b) a channel assignment game. We propose semi-distributed and distributed algorithms in order to find the Nash equilibrium for each game. We compare our proposals to the concurrent approaches in LoRaWAN networks and demonstrate the significant gains that they bring in terms of throughput, packet delivery ratios, and energy efficiency. 

## Figures and Tables

**Table 1:** LoRa in the 868 MHz band.

SF	Data Rate [kbps]	Sensitivity [dBm]	SNR [dB]
7	5.458	−123	[−7.5,*∞*]
8	3.125	−126	[−10,−7.5]
9	1.757	−129	[−12.5,−10]
10	0.976	−132	[−15,−12.5]
11	0.537	−134.5	[−17.5,−15]
12	0.293	−137	[−20,−17.5]
ϕ	0	Not covered	<−20

**Table 2:** Notation Summary.

Notation	Definition
$\lambda^{i}$	Packet generation rate for devices in network *i*
$T_{s}$	Time to transmit a packet on SF *s*
*l*	Packet length in bytes
*d*	Duty cycle
$N_{co}^{i}$	Number of covered nodes for operator *i*
$N_{s}^{i}$	Number of nodes that can use SF *s* and higher for operator *i*
$p_{s}^{i}$	Ratio of devices using SF *s* and belonging to operator *i*
*n*	Maximum number of channels that can be used by each operator
$G_{sc}$	Total normalized traffic load on SF *s* and channel *c*
$\lambda_{sc}^{e}$	Intensity of external traffic on SF *s* and channel *c*
N	Set of operators and *N* their number
T	Total normalized throughput
*S*	The number of available SFs

**Table 3:** Simulation Parameters.

Parameter	Value
Number of different networks	4
Number of devices per network	[750,1000,1250,1500] (4500 total)
Number of available channels	3 to 8
# of channels per operator *n*	1 to 3
Number of gateways per network	2
Network layout	Square with side *S* = 8 km
Path loss model	Okumura-Hata Model
Spreading factors SF	7–12
Tx power	14 dBm
Carrier frequency	868 MHz
Gateway height	30 m
Device height	1.5 m
Packet generation rate per network $\lambda^{i}$	[1,2,3,4] packets/hour
Packet length	50 Bytes
Duty Cycle	1%

## Data Availability

Data can be made available upon request.

## Abbreviations

The following abbreviations are used in this manuscript:

LoRaWAN	Long Range Wide Area Networks
SF	Spreading Factor
NE	Nash Equilibrium
ADR	Adaptive Data Rate
ISM	Industrial Scientific and Medical bands
